# Secondary Glaucoma Associated with Encircling Scleral Buckle Migration into the Cornea

**DOI:** 10.4274/tjo.02679

**Published:** 2016-01-05

**Authors:** Şengül Özdek, Murat Hasanreisoğlu, Ufuk Adıgüzel, Zeynep Aktaş

**Affiliations:** 1 Gazi University Faculty of Medicine, Department of Ophthalmology, Ankara, Turkey; 2 Mersin University Faculty of Medicine, Department of Ophthalmology, Mersin, Turkey

**Keywords:** Scleral buckle, migration, glaucoma

## Abstract

Transmuscular migration of the encircling band through rectus muscles and straddling of the cornea has only been reported in a few cases previously in the literature. This rare condition has never been associated with glaucoma. In this report, we aimed to describe a unique case with transmuscular migration of encircling buckle as a probable cause of glaucoma. A 17-year-old female presented with transmuscular migration of buckle and high intraocular pressure (IOP). Limbal/corneal migration of the silicone band was thought to be the main reason for the IOP rise; therefore, scleral band removal was performed. One month after removal, the patient was free of glaucoma medications and IOP was within normal limits. The retina remained attached during all postoperative visits. Transmuscular migration of the encircling band through rectus muscles and straddling of the cornea may act as a trigger for glaucoma.

## INTRODUCTION

Scleral buckling surgery is a commonly used surgical technique in the management of rhegmatogenous retinal detachment (RD). Extrusion, erosion, and intrusion of scleral buckling elements are rare yet significant complications of the scleral buckling procedure. These complications are more common with radial sponge material; however, they also occur as a rare complication with encircling silicone bands.^1^ Transmuscular migration of the encircling band through rectus muscles and straddling of the cornea has only been reported in a few cases previously in the literature.^2,3,4,5^ In this report, we aimed to describe a unique case with transmuscular migration of encircling buckle, which probably acted as a trigger for glaucoma.

## CASE REPORT

**Figure 1:**
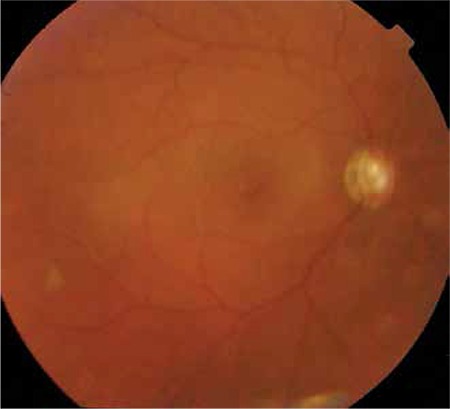
Fundus view of the patient after silicone oil removal. Slightly pale and tilted optic disc and attached retina can be seen. Note the fundus view is a bit cloudy because of the corneal scar

A 17-year-old female with vitreous hemorrhage and retinal detachment secondary to penetrating eye injury in the right eye underwent encircling silicone band surgery using 240S 2.4 mm silicone band together with pars plana vitrectomy, lensectomy, endolaser and silicone oil tamponade in October 2007. After surgery, the primary suturation zone of the penetrating injury appeared as a broad radial line extending from central cornea to peripheral infero-temporal zone. Iris loss in the same quadrant and aphakia were also noted. Glaucoma medications were administered for a short time after the surgery to prevent post-operative/traumatic intraocular pressure (IOP) spikes. Three weeks after the surgery, the patient was free of glaucoma medications and her IOP was 16 mmHg in that eye. The silicone oil was removed 3 months later and the retina was attached at all postoperative visits. Her corneal scarring, aphakia and slightly pale optic disc persisted (Figure 1). Her visual acuity was counting fingers from 4 meters with aphakic correction 3 months after silicone oil removal. IOP was 17 mmHg. She was discharged without any ocular medication and continued the follow-up visits in her hometown with her primary ophthalmologist.

**Figure 2:**
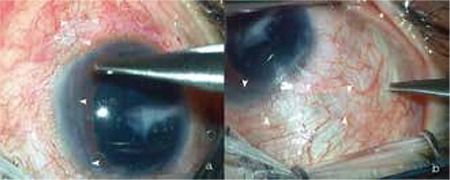
Anterior segment photos prior to silicone encircling band removal. Silicone band can easily be seen in the cornea at the nasal quadrant (a) and under the conjunctiva at the superotemporal quadrant (b) (White arrowheads)

Two years after silicone oil removal, the patient was referred again to our clinic because of intractable IOP increase. Her visual acuity was hand motions and IOP was 42 mmHg in the right eye. On slit-lamp examination, the most prominent finding in the anterior segment evaluation was the view of encircling silicone band at the nasal limbal intrastromal cornea and under the conjunctiva at the superior and inferior nasal quadrants (Figure 2a, 2b). There was no restriction in ocular movements. Gonioscopically, the band was seen to be positioned within the cornea and projected as a ridge into the anterior chamber with close proximity to the trabecular meshwork. No iris or angle neovascularization were observed. On fundoscopic examination, the retina was attached and the optic nerve was pale.

**Figure 3:**
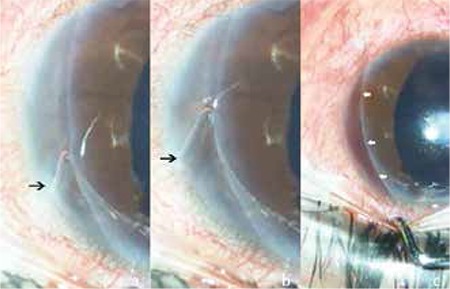
Silicone band leaving an intracorneal groove during removal from the cornea. The free edge of silicone band is marked with black arrows (a,b). Sero-hemorrhagic fluid filling the intrastromal groove after complete removal of silicone band (white arrows) (c)

Limbal/corneal migration of the silicone band was thought to be the main reason for the IOP rise; therefore scleral band removal was planned. Surgery was performed under local anesthesia. Inferotemporal conjunctival incision was used to reach the silicone band and the suture knot was cut to relieve the band. The sclera under the band was very necrotic and the choroid was clearly visible. The silicone band could easily be pulled out without any resistance and the movement of the band could be observed through the nasal limbal-corneal migration area (Figure 3a, 3b). After the removal, a mild leakage of sero-hemorrhagic fluid from the nasal iridocorneal angle filling the intrastromal groove was noticed (Figure 3c). The patient’s IOP in the right eye was 20 mmHg on the first postoperative day and fell to 16 mmHg within two weeks with anti-glaucoma topical medication. One month after the surgery the patient was again free of glaucoma medications and her IOP was 15 mmHg. The retina remained attached during all postoperative visits.

## DISCUSSION

Only a few cases of anterior transmuscular migration and intracorneal localization of the encircling band have been reported.^1,2,3,4,5^ The ‘cheesewiring’ process, which means spontaneous reattachment of muscle fibers to the sclera after erosion by the band, has been suggested to explain transmuscular migration. Similar to our case, in most of the cases no ocular motility problem has been observed.^1,2,3^ Kreis et al.^4^ reported the only case causing vertical diplopia. Saatci et al.^2^ reported a case of an encircling band causing corneal groove formation where the band was observed under intact corneal epithelium. The corneal groove disappeared following the band removal surgery. In another intracorneal encircling band case by Lopez et al.,^3^ the band was reported to be embedded deeper in the corneoscleral junction, and because the patient was asymptomatic, the encircling band was left at that position. In addition, Pearce and Roper-Hall5 reported a silicone strap case, eroded through the sclera and located intracorneally, which projected into the anterior chamber and caused iritis. None of those cases were determined to cause secondary glaucoma.

## CONCLUSION

In the presented case, severe IOP rise was observed after migration of the buckle. Due to the co-appearance of glaucoma and the intracorneal silicone band, the former was thought to be secondary to the latter. Therefore, the decision to remove the encircling band was made. The mechanism for IOP rise may be explained with the localization of the band at the deeper corneoscleral junction, which might narrow the iridocorneal angle and partially prevent aqueous humour out-flow. The almost immediate postoperative normalization of the patient’s IOP supports this idea. On the other hand, previous ocular trauma, presence of aphakia and previous vitrectomy could all be predisposing factors to IOP rise in this patient. Meticulous follow-up is crucial in order to observe any kind of secondary glaucoma and preserve the patient’s vision.

To the best of our knowledge, this is the first case of limbal intracorneal migration of encircling buckle as a cause of elevated IOP.

## Ethics

Informed Consent: It was taken.

Peer-review: Externally peer-reviewed.
